# Lightweight Visual Localization of Steel Surface Defects for Autonomous Inspection Robots Based on Improved YOLOv10n

**DOI:** 10.3390/s26072132

**Published:** 2026-03-30

**Authors:** Jinwu Tong, Xin Zhang, Xinyun Lu, Han Cao, Lengtao Yao, Bingbing Gao

**Affiliations:** 1 Machine Vision and Deformation Monitoring Laboratory, Artificial Intelligence and Precision Medicine Research Laboratory, Engineering Training Center, School of Applied Technology, Nanjing Institute of Technology, Nanjing 211167, China; y00450240449@njit.edu.cn (X.Z.); luxy@njit.edu.cn (X.L.); y00450240406@njit.edu.cn (H.C.); y00450240241@njit.edu.cn (L.Y.); 2 School of Automation, Northwestern Polytechnical University, Xi’an 710072, China; 3 Shenzhen Research Institute of Northwestern Polytechnical University, Shenzhen 518057, China

**Keywords:** deep learning, steel defect, image detection, YOLOv10n, visual positioning

## Abstract

To address the challenges of steel surface defect detection—characterized by fine-grained textures, substantial scale variations, and complex background interference—conventional lightweight detectors often struggle to balance real-time navigation requirements with high-precision spatial localization on mobile inspection platforms. In this work, we propose KDM-YOLO, a lightweight visual localization and detection method built upon YOLOv10n, designed to provide an efficient perception engine for autonomous inspection robots. The proposed approach enhances the baseline through three key perspectives: feature extraction, context modeling, and multi-scale fusion. Specifically, KWConv is introduced to strengthen the representation of fine-grained texture and edge cues; C2f-DRB is employed to enlarge the effective receptive field and improve long-range dependency perception to reduce missed detections; and a multi-scale attention fusion (MSAF) module is inserted before the detection head to adaptively integrate spatial details with semantic context while suppressing redundant background responses. Ablation studies confirm that each module contributes to performance gains, and their combination yields the best overall results. Comparative experiments further demonstrate that KDM-YOLO significantly improves detection performance while retaining a compact model size and high inference speed. Compared with the YOLOv10n baseline, Precision, Recall and mAP@50 are increased to 91.0%, 93.9%, and 95.4%, respectively, with a parameter count of 3.29 M and an inference speed of 155.6 f/s. These results indicate that KDM-YOLO achieves an ideal balance between the accuracy and computational efficiency required for embedded navigation platforms, providing an effective solution for online autonomous inspection and real-time localization of steel surface defects.

## 1. Introduction

With the in-depth development of Industry 4.0 and intelligent manufacturing, autonomous inspection robots equipped with visual perception have become the core tool for ensuring the reliability of modern industrial infrastructure. Steel, as a key basic structural material of modern industrial systems, is widely used in construction engineering, transportation equipment and high-end manufacturing [1,2]. The accurate identification and visual positioning of its surface defects are directly related to the accuracy of the robot’s subsequent autonomous maintenance, path planning and service reliability assessment.

The evolution of steel surface defect detection technology has roughly gone through three stages: manual inspection, rule-driven automated visual inspection and traditional machine learning methods. In the early stage, manual visual inspection was relied upon for experience-based judgment, which was easily affected by subjective factors and fatigue. Subsequently, the detection system gradually introduced non-destructive testing and traditional machine vision methods, and achieved defect detection and positioning through magnetic particle, penetrant, eddy current, ultrasonic and other detection methods, or combined with threshold segmentation, edge and texture analysis and other rule algorithms. However, these methods are highly sensitive to imaging conditions, and their robustness and positioning stability are severely restricted when mobile inspection platforms face complex and ever-changing production backgrounds. Research has introduced machine learning into the visual inspection process, forming a classic paradigm of “manual feature representation + classifier decision” [3].

In recent years, deep learning-led visual perception has significantly boosted the perception capabilities of autonomous inspection systems. Based on framework structure, existing methods mainly fall into two categories: two-stage and single-stage. Two-stage methods first locate potential defect regions, then perform category discrimination and boundary regression. Representative models include Faster R-CNN (Convolutional Neural Network) and its cascaded augmented forms (such as Cascade R-CNN). While they excel in localization accuracy, their long inference chain and high computational cost make it difficult to meet the stringent requirements of mobile robots for real-time navigation and vehicle power consumption. In contrast, single-stage methods, represented by the YOLO (You Only Look Once) series, simultaneously perform classification and regression on multi-scale feature maps. Their compact structure and fast inference speed allow sufficient computational resources for SLAM (Simultaneous Localization and Mapping) and path planning algorithms running simultaneously on inspection robots, making them more suitable for the online perception needs of high-speed production lines. Typical examples include SSD (Single Shot MultiBox Detector), RetinaNet, and the YOLO series, which often improve detection stability in complex backgrounds through multi-scale feature fusion, attention enhancement, and lightweight design. Based on the above context, this paper will summarize the representative models and key improvement ideas of the two types of frameworks, and analyze their applicability and limitations in the steel surface defect scenario.

Regarding the two-stage method, He et al. [4] constructed a steel surface defect detection framework based on Faster R-CNN, and proposed a multi-level feature fusion network (MFN) to integrate features from different stages of the backbone network across layers. This improves the detection capability of small defects and weak contrast defects while taking into account both high-level semantic discrimination and low-level localization details, and maintains good performance even when reducing the number of proposals. Damacharla et al. [5] proposed TLU-Net, which uses U-Net as the main body and ResNet/DenseNet pre-trained on ImageNet as the encoder for transfer learning initialization. At the same time, a linear multi-label classification head is introduced at the bottleneck feature to achieve collaborative learning of defect category discrimination and pixel-level segmentation, thereby enhancing the segmentation and recognition effect. Zhang et al. [6] used ResNet-50 as the backbone in the two-stage detection framework, introduced a convolutional block attention module to enhance the defect feature response, and adopted a guided anchoring region proposal mechanism to replace the traditional RPN in order to improve the quality of candidate regions and improve the sample allocation in the training stage. Wi et al. [7] proposed D^2^-SPDM, which uses the detection boxes generated by Faster R-CNN as weak supervision cues, first generates an initial mask, and then combines GrabCut and DeepLabv3+ for recursive learning to achieve pixel-level defect mapping with low annotation cost. Leng et al. [8] introduced a feature fusion module and a lightweight channel attention mechanism between the FPN and RPN of Faster R-CNN to address the problem of easily missed detection of subtle defects, so as to enhance the ability of multi-scale detail extraction and channel selection, thereby improving detection accuracy and robustness.

Although the two-stage method performs well in terms of localization accuracy and small defect detection, its inference process is multi-level and the computation and storage overhead is high, making it difficult to support real-time online detection of high-speed production lines in a long-term stable manner. Based on this, the research focus has gradually shifted to a more efficient single-stage paradigm. Among them, the YOLO series has become the mainstream framework for real-time detection of steel surface defects due to its end-to-end structure and good speed–accuracy trade-off. The improvement work around YOLO mainly focuses on feature representation enhancement, fusion path optimization and lightweight design: Li et al. [9] took YOLOv5s as the baseline, introduced a three-branch multi-scale feature extraction module (MSFE) and designed an efficient feature fusion module (EFF). At the same time, the Bottleneck structure was simplified and the backbone network was deepened to improve the efficiency of multi-scale expression and fusion. Yang et al. [10] proposed CFE-YOLOv8s, which realizes the local–global feature coupling of CNN-Transformer through CBS-BiFormer (CBiF), replaces the original C2f with Faster-C2f (FC) to reduce redundant computation, and embeds EMA in FC to build EFC to further enhance feature fusion. Chen et al. [11] improved YOLOv9 to address the problem of easily missed detection of small-scale defects. They introduced depthwise separable convolution (DSConv) to reduce complexity and combined C3, BiFPN, and DySample to enhance cross-layer fusion, multi-scale interaction and upsampling detail recovery, thereby improving the localization capability of small targets. Liao et al. [12] proposed YOLOv10n-SFDC, adding DualConv to the backbone to enhance the fusion of local details and cross-channel information. They replaced C2f with SlimFusionCSP in the detection head to achieve lightweight multi-scale fusion and used Shape-IoU loss to optimize the bounding box regression accuracy. Li et al. [13] built an improved model based on YOLOX, enriched gradient propagation through CSPCrossLayer, introduced Shuffle Attention (SA) to suppress background interference and highlight defect features, and used PSBlock in the fusion stage to reduce redundant computation and improve multi-scale fusion efficiency, thereby alleviating false detection and missed detection and improving detection accuracy.

Therefore, to address the challenges of spatial localization of fine-grained defects in complex environments and the limited computing resources faced by inspection robots, this paper proposes a lightweight perception method, KDM-YOLOv10n, for autonomous inspection and localization tasks. This method uses YOLOv10n as the baseline model and introduces a KernelWarehouse Convolution (KWConv) module in the downsampling stage of the backbone network. This module enhances edge feature extraction through dynamic convolutional kernel combinations, improving the sharpness of defect target localization in complex backgrounds. Furthermore, the original C2f module is specifically improved at the key feature layer by introducing a C2f-DRB structure to enhance the network’s ability to express complex backgrounds and fine-grained defect features. This avoids introducing redundant computations in shallow and deep features and strengthens the capture of large-scale and long-distance dependent features, reducing target omissions during inspection. A multi-scale attention fusion module (MSAF) is introduced before the detection head to adaptively allocate weights and fuse information for detection features at different scales, enhancing cross-scale feature interaction capabilities and thus improving the detection accuracy of small-sized and complex-shaped defects. Through the above improvements, KDM-YOLOv10n significantly enhances the collaborative performance of target recognition and spatial positioning while maintaining its lightweight characteristics, providing efficient visual perception support for the autonomous online inspection of industrial robots.

## 2. Basic Principles

### 2.1. Overview of the YOLOv10n Algorithm

YOLOv10 is a one-stage object detection algorithm released by the Ultralytics team, and it can be extended to multiple vision tasks such as multi-object detection, instance segmentation, object tracking, and classification [14]. The YOLOv10 family provides five model scales—YOLOv10n, YOLOv10s, YOLOv10m, YOLOv10l, and YOLOv10x—so that users can choose an appropriate trade-off between accuracy and efficiency for different application requirements. As the lightweight variant, YOLOv10n offers clear advantages in model size, computational cost, and inference speed, enabling stable detection performance while maintaining high efficiency.

In terms of the detection pipeline, YOLOv10 introduces a dual-assignment strategy that fundamentally reduces the dependence on non-maximum suppression (NMS), thereby enabling an end-to-end detection paradigm without additional post-processing. This design not only improves overall inference efficiency but also alleviates false detections that may arise from candidate box filtering in dense or heavily occluded scenarios. Moreover, YOLOv10 adopts Selective Channel Downsampling (SCDown), which decouples the compression of spatial and channel dimensions to reduce feature redundancy while preserving critical information, leading to stronger feature representations in complex scenes [15]. In addition, a Position-Sensitive Attention (PSA) module is incorporated to model spatial positional cues within feature maps and emphasize informative regions, producing more discriminative features for cluttered backgrounds and small-object detection tasks.

**Figure 1:**
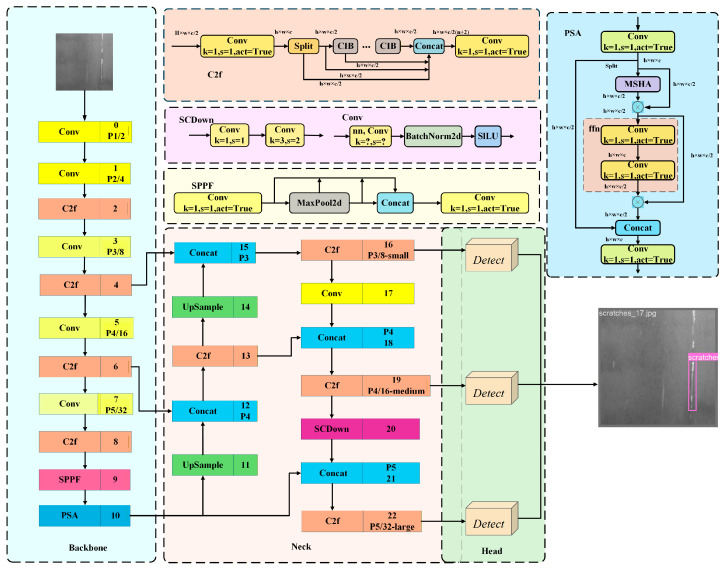
Architecture of the YOLOv10n network model.

The YOLOv10 architecture consists of three main components: a backbone network (Backbone), a neck network (Neck), and a head network (Head) [16], as illustrated in Figure 1.

### 2.2. Improvements to the YOLOv10n Algorithm

**Figure 2:**
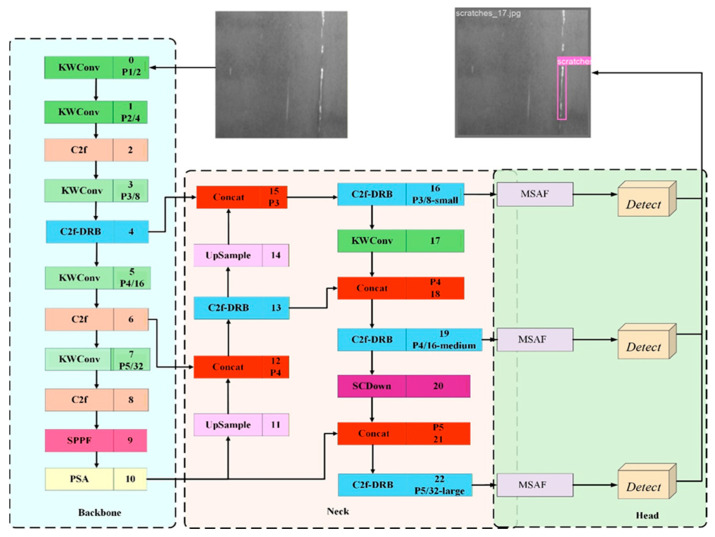
Network architecture of KDM-YOLO.

In this paper, we propose KDM-YOLO, a lightweight defect detection method built on YOLOv10n. Specifically, KWConv is introduced to enhance the representation of fine-grained texture and edge features; C2f-DRB is adopted to enlarge the effective receptive field and strengthen long-range dependency modeling, thereby reducing missed detections; and a multi-scale attention fusion module (MSAF) is inserted before the detection head to adaptively integrate spatial details with semantic context while suppressing redundant background responses. The overall architecture of KDM-YOLO is illustrated in Figure 2.

#### 2.2.1. KWConv Module

**Figure 3:**
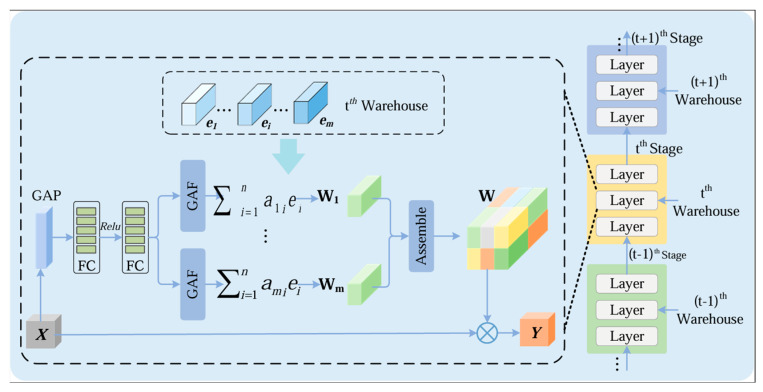
Structure of KWConv.

KernelWarehouse Convolution (KWConv) is an improved convolutional structure based on the concepts of kernel sharing and dynamic combination. Its core objective is to enhance parameter utilization efficiency and feature representation capacity while preserving the standard computational form of convolution. Unlike conventional convolution layers that assign a fixed set of kernels to each layer, KWConv constructs a shared Kernel Warehouse, where multiple kernels are stored as reusable basic units and are adaptively combined according to the input features, enabling more flexible feature modeling. The structure of KWConv is illustrated in Figure 3.

KWConv consists of two components: a kernel warehouse and a weight generation mechanism. The kernel warehouse stores multiple groups of base convolution kernels that are shared across different input channels or network layers. The weight generation mechanism produces input-adaptive combination weights from the current feature maps, which are then used to perform a weighted aggregation of the base kernels in the warehouse. In this way, KWConv enables the dynamic construction of diverse effective kernels without noticeably increasing the number of parameters.

For the feature maps output by each layer of the model, their spatial dimensions are uniformly represented as C×H×W. Here, C (Channels) represents the number of feature channels, indicating the depth of semantic features extracted by the model at different levels; H (Height) and W (Width) represent the spatial height and width of the feature map, respectively. These dimensions collectively determine the information capacity of the feature tensor in the network. Let the input feature map be $X\in R_{C\times H\times W}$, and assume the kernel warehouse contains N base convolution kernels, denoted as { $K_{1}$, $K_{2}$, …, $K_{N}$}. For a given input feature, KWConv first obtains the corresponding weighting coefficients $\alpha_{i}$ through a weight generation function. The resulting effective convolution kernel $K_{eff}$ for convolution can be expressed as:

(1) $$K_{eff}=\sum_{i=1}^{N}\alpha_{i}K_{i}$$

where $\alpha_{i}$ denotes the combination weight of the i-th base kernel. The output feature map Y is then obtained via a standard convolution operation:

(2) $$Y=X\times K_{eff}$$

In object detection tasks—particularly in industrial scenarios such as steel surface defect inspection—targets often exhibit large scale variations, complex shapes, and weak texture cues. By incorporating kernel sharing and dynamic combination, KWConv enables the network to more effectively exploit feature information across different scales and morphologies, thereby enhancing feature extraction capability and detection robustness under complex backgrounds. Consequently, integrating KWConv into a lightweight detection network helps improve overall detection performance while maintaining computational efficiency.

#### 2.2.2. C2f-DRB

**Figure 4:**
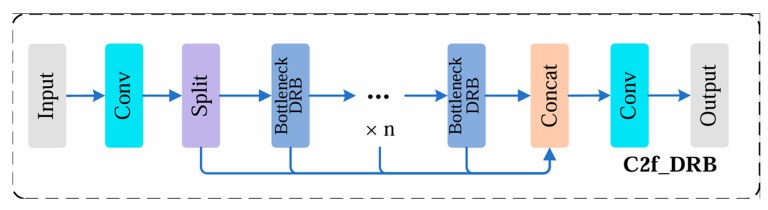
Structure of C2f-DRB.

The conventional C2f module achieves efficient feature propagation through cross-stage partial connections. However, under complex backgrounds and small-target scenarios, the original C2f still exhibits limitations in modeling contextual information and multi-scale features. This is particularly evident in steel surface defect detection, where defect appearances can differ substantially. For instance, scratches and pits often present more salient local patterns, while different defect types may also span wide spatial regions, resulting in long-range structural characteristics. Therefore, to enhance the multi-scale representation capability of the YOLO architecture while preserving lightweight design requirements, this paper introduces a feature enhancement unit based on a dilated residual block into selected C2f modules of YOLOv10n, forming the proposed C2f-DRB module. The structure of C2f-DRB is illustrated in Figure 4.

For large-area steel surface defect inspection, large convolution kernels are often beneficial because they can capture broader contextual information. However, directly using large kernels may weaken the emphasis on the kernel’s central region, which can adversely affect the extraction of fine details and critical features. Introducing a Dilated Residual Block (DRB) can alleviate this issue to a certain extent. Compared with the original module, DRB is designed such that, during inference, only a single convolution is required to approximate the feature extraction effect of multiple parallel convolutions, thereby reducing computational cost. Specifically, in the large-kernel setting, DRB employs several parallel convolution branches during training, but merges them into an equivalent single convolution at inference time. This re-parameterization strategy substantially lowers computation while preserving the benefits of multi-branch feature aggregation. In addition, repeated convolutional operations within the kernel center and its neighborhood accumulate stronger effective weights, enlarging the receptive field and enabling more accurate capture of key defect cues. As a result, compared with the original C2f, C2f-DRB effectively provides a larger receptive field and stronger central-feature emphasis, making it more suitable for steel surface defect detection.

In implementation, while retaining the cross-stage partial connection structure of the original C2f, the standard Bottleneck is replaced with a Bottleneck-DRB that incorporates dilated convolution. Within the C2f architecture, multiple Bottleneck-DRB units are stacked to progressively expand the receptive field and achieve effective multi-level feature fusion.

#### 2.2.3. Multi-Scale Attention Fusion (MSAF) Module

**Figure 5:**
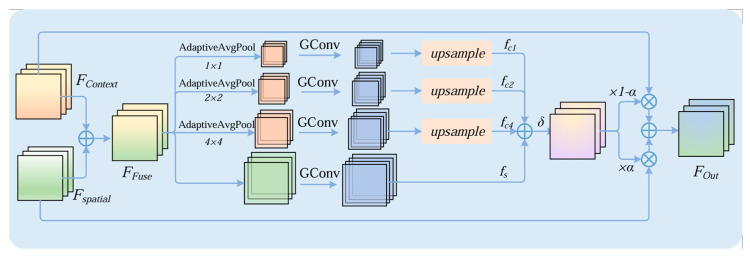
Structure of MSAF.

Steel surface defects exhibit substantial variations in scale, shape, and texture; therefore, features from a single level often struggle to simultaneously capture fine-grained texture details and high-level semantic context. To strengthen information flow across different layers and alleviate the attenuation of shallow details during deep feature propagation, this paper introduces a Multi-Scale Attention Fusion (MSAF) module before the detection head to improve the model’s adaptability to defect scale variations. The structure of MSAF is shown in Figure 5.

Structurally, MSAF can be divided into two stages: multi-scale attention modeling (MSA) and attention-guided fusion (Fusion). In the first stage, MSA estimates feature importance at different scales and outputs attention weights. In the second stage, Fusion uses the attention weights produced in the first stage to perform weighted integration of two complementary feature representations, yielding the final fused output. In our network, MSAF is placed before the Detect module on the three detection branches to enhance and re-fuse detection features at different scales.

In the multi-scale attention modeling (MSA) stage, let the input feature from the neck network be $F_{Fuse}\in R_{C\times H\times W}$. MSA generates a weight map α through two branches: region attention and pixel attention.

In the region-attention branch, a coarse-to-fine partitioning strategy is adopted to capture statistical cues at different granularities. Specifically, the feature map is divided into multiple grid scales (e.g., 1 × 1, 2 × 2, and 4 × 4). Average pooling is applied within each grid cell to obtain regional descriptors, which are then passed through convolution layers for channel compression and restoration. The resulting features are finally upsampled to the original resolution to align with the outputs of other branches. This process introduces multi-granularity contextual information while keeping the computational overhead under control.

For any grid scale s ∈ S (where S = {1, 2, 4}), the process can be formulated as:

(3) $$R_{S}=U\left(\Phi \left(\psi \left(AvgPool_{s}\left(F_{Fuse}\right)\right)\right)\right)$$

where AvgPools denotes average pooling over an s × s grid, ψ and ϕ represent the convolutional mappings for channel compression and restoration, respectively, and U denotes upsampling (or unpooling) back to H × W. The final output of the region branch is given by:

(4) $$R=\sum_{s\in S}R_{s}$$

In the pixel-attention branch, a pixel-wise attention map is generated by applying a lightweight transformation to $F_{Fuse}$, producing a response map $P\in R_{C\times H\times W}$ with the same spatial resolution as the input.

Then, MSA sums the outputs of the two branches and applies a sigmoid function to obtain the attention weight map:

(5) $$\alpha =\sigma (R+P),\alpha \in R_{C\times H\times W}$$

where σ denotes the sigmoid activation function.

Finally, in the fusion stage, MSAF introduces two complementary feature streams: a context feature $F_{context}\;$ and a spatial feature $F_{spatial}$. These features may originate from different sources (e.g., a context branch formed by aligning adjacent-layer features and a spatial branch that preserves fine details at the current layer), or they can be obtained by applying two lightweight convolutional transformations to $F_{Fuse}$. This design makes the module plug-and-play and suitable for lightweight detection networks. The fused output is gated by the attention weights α, yielding the final feature $F_{out}$:

(6) $$F_{out}=F_{context}\alpha +F_{spatial}(1-\alpha )$$

The key role of MSAF is to leverage MSA to establish complementary constraints between multi-granularity regional statistics and pixel-level responses, producing an interpretable weight map α. This weight map is then used to adaptively balance contextual and spatial information, resulting in more discriminative multi-scale fused features before the detection head.

## 3. Experimental Dataset and Experimental Environment

### 3.1. Dataset Collection and Preprocessing

The steel surface defect dataset used in this study, NEU-DET, was collected by a research team from Northeastern University [17]. It contains six categories of steel surface defects: Rolled-in Scale (RS), Patches (Pa), Crazing (Cr), Pitted Surface (PS), Inclusion (In), and Scratches (Sc). The dataset comprises 1800 images in total, with 300 grayscale images per defect category. All images have a resolution of 200 × 200, and the six categories share comparable image quality. The dataset is split into training, validation, and test sets at a ratio of 8:1:1.

### 3.2. Experimental Setup

The experiments were conducted on a Windows 11 platform. The CPU was an AMD Ryzen 9 7945HX, and the GPU was an NVIDIA GeForce RTX 4060, with 32 GB of system memory. Detailed hardware specifications are provided in Table 1. During training, the number of epochs was set to 200, the batch size was 16, the learning rate was 0.01, and the momentum was 0.937. The SGD optimizer was employed, and the detailed hyperparameter settings are listed in Table 1. To ensure fairness and consistency in the evaluation, all comparative experiments were conducted within a unified system architecture. During the inference phase, all models were tested in their original PyTorch format (.pt), without using ONNX, TensorRT, or other additional quantization acceleration methods. This setup aims to evaluate the models’ raw computational efficiency and generalization performance within a general deep learning framework, thus providing a basic reference for practical deployment.

## 4. Experimental Results and Analysis

### 4.1. Evaluation Indicators

To accurately evaluate the detection accuracy and inference efficiency of the proposed method for steel surface defects, Precision (P), Recall (R), and mean Average Precision (mAP) are adopted as evaluation metrics. Their formulations are given as follows:

(7) $$P=\frac{TP}{TP+FP}$$

(8) $$R=\frac{TP}{TP+FN}$$

where TP denotes the number of samples for which the defect regions are correctly detected, FP represents the number of non-defective samples that are incorrectly predicted as defective, and FN indicates the number of defective samples that are missed by the detector.

Because steel surface defect detection involves multiple defect categories, Precision and Recall alone cannot comprehensively reflect overall model performance. Mean Average Precision (mAP) is a core evaluation metric in the field of object detection. In this study, we primarily use mAP@50, which is the average precision when the Intersection over Union (IoU) threshold is set to 0.5. This means that a prediction is considered a True Positive only when the overlap area between the predicted bounding box generated by the model and the ground truth bounding box reaches or exceeds 50%. This metric balances detection sensitivity and localization accuracy and is a commonly used benchmark for evaluating the robustness of industrial defect detection algorithms. During training, a corresponding PR curve can be obtained for each defect category, and the area under the curve is used to compute the mAP, which can be expressed as:

(9) $$AP=\int_{0}^{1}P(R)dR$$

(10) $$mAP=\sum_{i=1}^{N}\frac{AP(i)}{N}$$

### 4.2. Ablation Experiment

To systematically evaluate the effectiveness of each proposed module, ablation experiments were conducted using YOLOv10n as the baseline. Under the same dataset split and training settings, KWConv, C2f-DRB, and MSAF were introduced step by step. The resulting changes in Precision, Recall, and mAP@50, as well as model size and inference speed, were comprehensively compared. The experimental results are summarized in Table 2.

The baseline YOLOv10n achieves P = 80.3%, R = 81.7%, and mAP@50 = 88.0% on the test set, with 2.77 M parameters and an inference speed of 178.2 f/s. Although the baseline model is lightweight and fast, it still suffers from false positives and missed detections under complex textured backgrounds. After introducing KWConv alone, the convolutional representation captures fine-grained textures and edge cues more effectively, improving the separability between defects and background; consequently, P, R, and mAP@50 increase simultaneously, while the changes in model size and speed remain marginal. Replacing the original module with C2f-DRB further boosts performance: the dilated residual design enlarges the effective receptive field and strengthens long-range contextual dependencies, enhancing the continuous response to elongated, discontinuous, or low-contrast defects. As a result, missed detections are reduced markedly, leading to a more pronounced gain in Recall and a further increase in mAP@50, with a still-controlled parameter growth. When only MSAF is added, explicit attention weights are used to gate and fuse spatial details with contextual features, suppressing background interference and redundant activations. This yields a noticeable improvement in Precision and a clear mAP gain; however, due to attention gating and multi-branch computation, the inference speed decreases more evidently, and Recall may fluctuate under a fixed confidence threshold.

When KWConv and MSAF are combined, the model further improves to P = 90.6% and R = 90.1%, reaching mAP@50 = 95.0%, with 3.20 M parameters and 157.8 f/s, which is almost the same speed as using MSAF alone. This suggests that the two modules are complementary: KWConv strengthens fine-grained feature extraction, while MSAF adaptively re-weights multi-scale features during fusion, reinforcing clearer defect responses across layers and further suppressing background noise. Therefore, Recall rebounds while Precision remains high, pushing mAP@50 higher, and the additional overhead introduced by KWConv is limited. With all three modules enabled, the model attains the best overall performance (P = 91.0%, R = 93.9%, mAP@50 = 95.4%), with only a slight increase in parameters to 3.29 M and a still-high speed of 155.6 f/s.

These results indicate that the three modules form a synergistic pipeline across “representation enhancement–receptive-field expansion–fusion selection”: KWConv improves local texture discrimination, C2f-DRB strengthens cross-region contextual modeling and substantially reduces missed detections, and MSAF further performs attention-guided selective fusion of multi-scale information to suppress false positives and redundant responses. Consequently, Recall achieves the largest gain and drives mAP@50 to the highest level. Although the inference speed decreases compared with the baseline, the model remains lightweight and maintains high throughput, satisfying real-time deployment requirements.

### 4.3. Comparative Experiment

As shown in Table 3, the proposed KDM-YOLO demonstrates a significant leading position in several key performance indicators. Compared to the baseline YOLOv10n model, KDM-YOLO achieves a leap in detection accuracy with only a minor increase in the number of parameters from 2.77 M to 3.29 M. Specifically, the precision reaches 91.0%, the recall jumps dramatically from 81.7% to 93.9%, and the mAP@50 metric ultimately reaches 95.4%. This growth fully validates the effectiveness of the feature enhancement strategy designed in this paper in handling complex industrial contexts. Although the inference speed decreases from 187.2 f/s to 155.6 f/s, this speed is still sufficient to support the stringent real-time requirements of various mobile inspection platforms, achieving a trade-off between minimal computational resources and significant accuracy gains.

In comparisons with current mainstream algorithms and various improved versions of YOLO, KDM-YOLO demonstrates outstanding parameter efficiency and detection performance. For example, while CK-NET achieves an average accuracy of 92.1%, its model has a massive 24.4 M parameters, while KDM-YOLO requires only about one-eighth of those parameters to surpass it in accuracy by 3.3 percentage points. When facing recent improvements such as YOLOv8n-SDEC, DDI-YOLO, and ESE-YOLO, KDM-YOLO maintains a lightweight design while exhibiting an overwhelming advantage in the core metric of mAP@50, with an accuracy lead of up to approximately 19 percentage points. This advantage ensures that the model can still provide highly reliable visual perception support in resource-constrained embedded environments.

KDM-YOLO demonstrates extremely powerful feature capture capabilities for cracking defects, the most difficult type of defect to detect on steel surfaces. Experimental data show that the baseline YOLOv10n has an average accuracy of only 0.557 in this category, while this paper successfully improves the accuracy to 0.805 by using KWConv to enhance edge extraction and MSAF module to achieve adaptive multi-scale fusion. This ability to detect subtle targets with highly interfering backgrounds makes KDM-YOLO extremely valuable in industrial inspection tasks where false negative rates are critical.

### 4.4. Visualization Analysis

**Figure 6:**
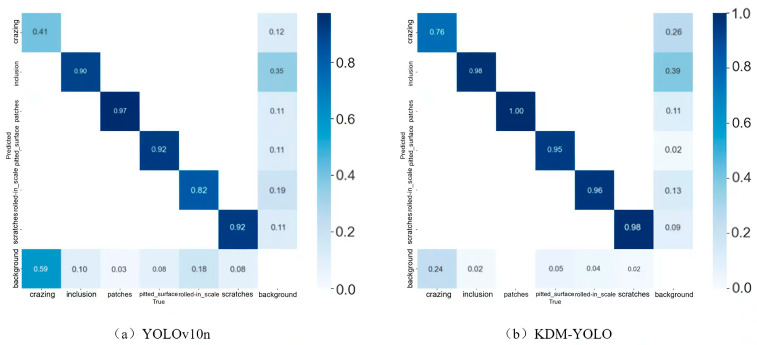
Confusion matrix comparison chart.

To visually demonstrate the classification and discrimination capabilities of KDM-YOLO in steel surface defect identification, this paper compares the normalized confusion matrices of KDM-YOLO with the YOLOv10n baseline model, as shown in Figure 6. The confusion matrix uses the true class as the horizontal axis and the predicted class as the vertical axis. The main diagonal elements reflect the proportion of correctly identified categories, while the off-diagonal areas represent the distribution of misclassifications. The comparison shows that the main diagonal area of KDM-YOLO is generally darker in color and has higher corresponding values, indicating that its consistency and accuracy in identifying various defects are superior to the baseline model. Simultaneously, the proportion of easily confused samples judged as background has decreased, especially in categories with complex and difficult-to-distinguish textures such as crazing, demonstrating a more significant improvement. This indicates that the proposed method effectively reduces false negatives and false positives, thereby improving overall detection performance.

**Figure 7:**
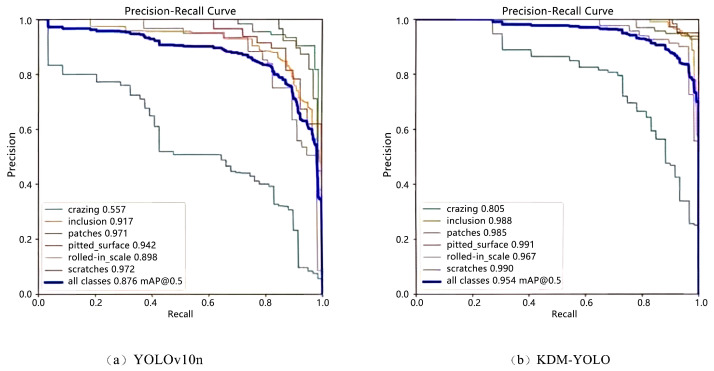
Comparison of P-R curves.

To further compare the detection capabilities of the models at different confidence thresholds, Figure 7 shows a comparison of the PR curves of YOLOv10n and KDM-YOLO. It can be seen that the overall PR curve of KDM-YOLO is closer to the upper right corner, with a larger curve envelope area. The corresponding overall mAP@0.5 increases from 0.876 to 0.954, indicating a better performance in the trade-off between precision and recall. Looking at the categories, the performance bottleneck of the baseline model is mainly concentrated in the crazing class, where precision decreases significantly during recall improvement, with an AP of only 0.557. After introducing KDM improvements, the AP for this class increases to 0.805, indicating that the model’s ability to distinguish fine texture defects is significantly enhanced, and false negatives and missed detections are effectively suppressed. Meanwhile, the AP values for inclusion and rolled-in scale categories increased from 0.917 to 0.988 and from 0.898 to 0.967, respectively. The AP values for other categories also remained at a high level and further improved, demonstrating KDM-YOLO’s stable detection capability and overall robustness in complex backgrounds.

**Figure 8:**
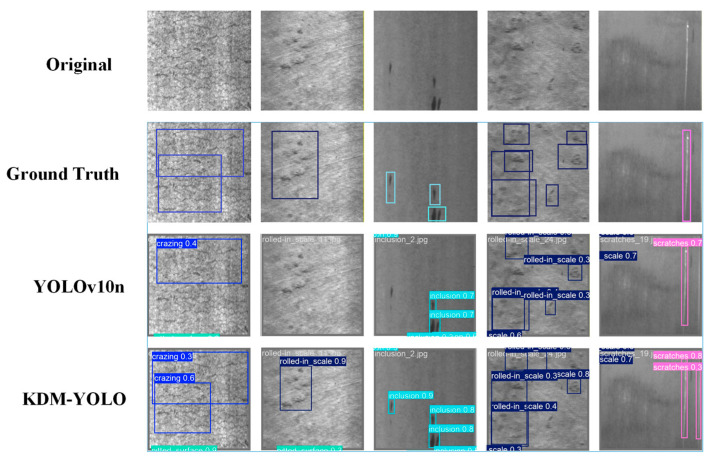
Comparison of detection performance between YOLOv10n and KDM-YOLO models.

In the qualitative visualization comparison, Figure 8 shows the detection results of the same set of samples in three scenarios: the original image, YOLOv10n, and KDM-YOLO. For defects with significant scale variations or discrete distributions, such as rolled-in scale and inclusion, KDM-YOLO’s detection bounding box coverage is closer to the defect subject, and its detection of similar defects is more stable, indicating that it is more effective in improving the separability of targets in terms of multi-scale feature fusion and context modeling. For slender scratch-like targets, KDM-YOLO can detect strip-shaped defects more fully and improve confidence while maintaining reasonable localization, indicating that its ability to perceive long-distance dependence and local continuous texture is enhanced. Overall, this comparison figure intuitively verifies that KDM-YOLO has better detection completeness and prediction reliability than YOLOv10n for difficult-to-detect categories in complex backgrounds, which is consistent with the improvement trend of the aforementioned quantitative indicators Precision, Recall, and mAP@50.

**Figure 9:**
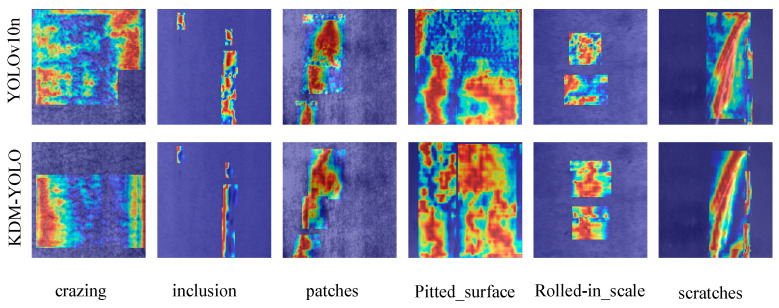
Comparison of YOLOv10n and KDM-YOLO heatmaps.

Figure 9 shows a comparison of the EigenCAM heatmaps of the native YOLOv10n and the improved KDM-YOLO model presented in this paper on typical samples of the NEU-DET dataset.

Observing the performance of the baseline models in the first row of Figure 9, it can be seen that the native YOLOv10n generally exhibits a significant diffusion phenomenon in its feature response regions when identifying defects on steel surfaces. Especially when facing finely textured pitted surfaces or minor cracks, the thermally highlighted areas generated by the model often fail to accurately pinpoint the defect core, instead diffusing into the background area surrounding the target, accompanied by obvious irrelevant activations. This phenomenon indicates that the baseline model has limited ability to identify key semantic features of defects under complex texture interference, and its decision-making process is easily misled by background noise, leading to deviations in localization accuracy.

In contrast, the KDM-YOLO model shown in the second row of Figure 9 achieves a significant improvement in feature focusing ability. For defects with clear linear geometric properties, such as scratches and inclusions, the improved model’s thermal distribution closely matches the true physical contours of the defects, exhibiting highly concentrated feature response centers with clear edge boundaries. Furthermore, when dealing with large-area defects with irregular shapes, such as rolled-in scale, KDM-YOLO not only maintains the coherence and integrity of the attention region but also effectively suppresses ineffective activation of background-irrelevant textures. This evolution from fuzzy to precise directly verifies the effectiveness of the proposed KDM module in optimizing feature weights and enhancing spatial discriminative power. Visual evidence strongly demonstrates that the improved model can eliminate environmental interference and accurately focus on the most identifiable defect feature regions, thus fundamentally ensuring the reliability of detection results and the interpretability of the model in complex industrial scenarios.

### 4.5. Generalization Experiment

To deeply evaluate the robustness and feature transfer capability of the proposed KDM-YOLO model in unpredictable scenarios, this study introduces a third-party bearing surface defect dataset independent of the original training domain for generalization validation experiments. This dataset covers eight typical bearing manufacturing defects, including Casting_burr, Crack, Scratch, and Strain. Because this dataset is collected from different industrial inspection environments, its imaging background, illumination distribution, and target geometry all exhibit significant domain shifts compared to the initial training steel surface dataset. Under zero-shot inference conditions without any fine-tuning of the target domain data, comparing the accuracy performance of the baseline model and the improved model allows for an objective evaluation of the algorithm’s universality in handling unknown interference features.

**Figure 10:**
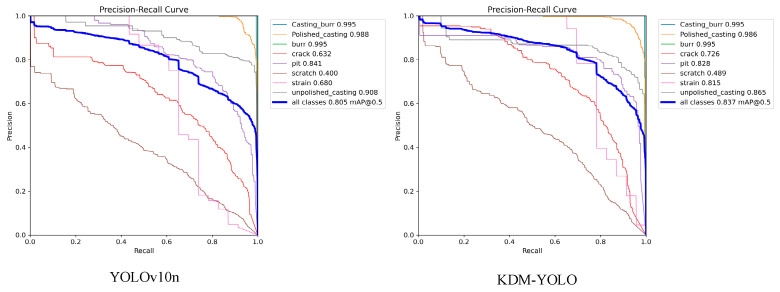
Comparison of Precision-Recall curves on a bearing surface defect generalization dataset.

The experimental results are shown in Figure 10, with quantitative indicators clearly revealing the superior performance of the improved model in cross-domain tasks. The baseline YOLOv10n model achieved a mean accuracy (mAP@0.5) of 0.805 on the new bearing test set, demonstrating a certain feature capture capability. The improved KDM-YOLO model significantly improved this to 0.837, achieving a 3.2% accuracy leap. This overall performance improvement strongly demonstrates the effectiveness of the proposed KDM-YOLO model in feature space reconstruction and core semantic enhancement, proving that the model is not merely overfitting the source domain data, but truly extracts common defect features with universal discriminative significance.

A deeper analysis of the precision-recall curves for different categories reveals that KDM-YOLO exhibits extremely strong robustness against interference when handling “weak classes” in the generalization experiment. Specifically, the baseline model performed poorly when dealing with the Scratch category under complex bearing textures, with an AP value of only 0.400, while KDM-YOLO optimized this metric to 0.489. In categories such as Crack and Strain, the improved model achieved significant improvements of 9.4% and 13.5%, respectively. This significant performance gain indicates that the KDM module can effectively guide the network to accurately locate defects even in unfamiliar background environments, effectively suppressing false detections caused by cross-domain noise. In summary, the generalization experiment results not only affirm the high accuracy characteristics of KDM-YOLO but also verify its high practical transfer value in complex and ever-changing industrial vision inspection tasks.

## 5. Conclusions

This paper addresses key challenges faced by autonomous inspection robots in locating defects on steel surfaces, including large defect scale spans, complex textures leading to difficulty in localization, and limited computing power of navigation platforms. Based on YOLOv10n, a lightweight perception model, KDM-YOLO is constructed and proposed. This method significantly improves the robot’s target localization accuracy during movement through three dimensions of improvement. First, the introduction of KWConv enhances the model’s ability to characterize fine-grained textures and defect edges, effectively improving the sharpness of target boundary localization in complex backgrounds. Second, the application of a C2f-DRB structure in the backbone and neck network expands the effective receptive field, enhancing long-distance dependent perception and improving the continuous response to slender or weak-contrast defects, thereby reducing the target mislocalization rate in the inspection path. Finally, an MSAF module is integrated in front of the inspection head to achieve attention-guided multi-scale feature fusion. This effectively suppresses background noise in the industrial environment while enhancing the interaction of cross-layer information, ensuring stable spatial localization performance for the robot at different observation distances. Experimental results show that KDM-YOLO significantly improves upon the YOLOv10n baseline in terms of Precision, Recall, and mAP@50, meeting the real-time requirements of the perception module in mobile robot navigation systems and reserving sufficient computational space for the underlying path planning algorithm. Visual analysis further confirms that KDM-YOLO exhibits higher consistency for difficult-to-detect categories, effectively mitigating false alarms and missed alarms during navigation. In summary, the proposed perception scheme achieves high-performance detection with low computational overhead, demonstrating significant engineering application value and deployment potential in the fields of autonomous inspection and visual localization of industrial robots.

For minor defects (such as fine cracks and scattered pits) in complex industrial scenarios, the KDM-YOLO proposed in this study shows good small target perception ability. By dynamically adjusting the local feature weights through the KDM module, the model can capture subtle feature changes at the sub-pixel level, thus effectively solving the problem that small targets are easily lost in deep convolutional networks. This robust ability to capture small targets is not only crucial in industrial quality inspection, but also has great application potential in fields sensitive to small targets, such as sports analysis [24] and remote sensing image analysis [25].

While KDM-YOLO has demonstrated superior performance in various industrial defect detection tasks, it still has certain limitations under specific extreme conditions. For example, on metal surfaces with extremely low lighting or strong specular reflection, the model’s feature extraction capabilities may be affected by environmental noise, leading to missed detections of weak features. When extremely high-density overlapping defects occur in industrial settings, the lightweight architecture may also face challenges in handling complex spatial topologies. Furthermore, although KDM-YOLO boasts extremely high inference efficiency, achieving ultra-real-time inference on very low-end embedded devices without GPU acceleration (such as low-computing-power microcontrollers) it still requires further algorithm pruning or quantization. Future research will focus on introducing adaptive lighting enhancement modules and exploring the model’s generalization performance on a wider range of material surfaces.

## Figures and Tables

**Table 1:** Experimental environment.

Environmental Parameters	Environment Configuration
CPU	R9-7945HX
GPU	NVIDIA GeForce RTX4060
GPU memory	32 G
operating system	Windows10
CUDNN	8.5.0
CUDA	11.8
Python	3.11
Pytorch	2.0.1

**Table 2:** Comparison of ablation test results.

Experiment	Model	P (%)	R (%)	mAP@50 (%)	Params (M)	FPS (f/s)
1	YOLOv10n	80.3	81.7	88.0	2.77	178.2
2	YOLOv10n + KWConv	85.9	87.2	92.2	2.83	171.3
3	YOLOv10n + C2f-DRB	88.5	90.4	94.7	2.87	165.2
4	YOLOv10n + MSAF	90.3	88.7	94.0	3.15	158.9
5	YOLOv10n + KWConv + MSAF	90.6	90.1	95.0	3.20	157.8
6	YOLOv10n + KWConv + C2f-DRB + MSAF	91.0	93.9	95.4	3.29	155.6

**Table 3:** Performance comparison of different models.

Model	P (%)	R (%)	mAP@50 (%)	Params (M)	FPS (f/s)
YOLOv5	85.7	74.1	77.9	10.3	95.2
YOLOv8	79.3	82.9	82.4	3.1	99
YOLOv11n	91.9	80.7	91.8	2.5	176
YOLOV8n-SDEC [18]	71.2	71.3	76.7	4.9	303
PIC2f-YOLO [19]	-	-	78.0	2.6	82
YOLOv8n-CSG [20]	-	73.0	76.8	1.95	-
DDI-YOLO [21]	72.5	71.4	78.3	2.66	158
ESE-YOLO [22]	-	-	76.3	2.3	-
CK-NET [23]	-	-	92.1	24.4	-
YOLOv10n	80.3	81.7	88.0	2.77	187.2
KDM-YOLO	91.0	93.9	95.4	3.29	155.6

## Data Availability

The NEU-DET dataset used in this study is publicly available at https://www.kaggle.com/datasets/kaustubhdikshit/neu-surface-defect-database (accessed on 15 December 2025).
